# Current perspectives on diffuse midline glioma and a different role for the immune microenvironment compared to glioblastoma

**DOI:** 10.1186/s12974-022-02630-8

**Published:** 2022-11-19

**Authors:** Casper J. Pachocki, Elly M. Hol

**Affiliations:** Department of Translational Neuroscience, University Medical Center Utrecht Brain Center, Utrecht University, Utrecht, The Netherlands

**Keywords:** Diffuse midline glioma, Diffuse intrinsic pontine glioma, Glioblastoma, Immune microenvironment, Tumor-associated microglia/macrophages, H3K27M

## Abstract

Diffuse midline glioma (DMG), formerly called diffuse intrinsic pontine glioma (DIPG), is a high-grade malignant pediatric brain tumor with a near-zero survival rate. To date, only radiation therapy provides marginal survival benefit; however, the median survival time remains less than a year. Historically, the infiltrative nature and sensitive location of the tumor rendered surgical removal and biopsies difficult and subsequently resulted in limited knowledge of the disease, as only post-mortem tissue was available. Therefore, clinical decision-making was based upon experience with the more frequent and histologically similar adult glioblastoma (GBM). Recent advances in tissue acquisition and molecular profiling revealed that DMG and GBM are distinct disease entities, with separate tissue characteristics and genetic profiles. DMG is characterized by heterogeneous tumor tissue often paired with an intact blood–brain barrier, possibly explaining its resistance to chemotherapy. Additional profiling shed a light on the origin of the disease and the influence of several mutations such as a highly recurring K27M mutation in histone H3 on its tumorigenesis. Furthermore, early evidence suggests that DMG has a unique immune microenvironment, characterized by low levels of immune cell infiltration, inflammation, and immunosuppression that may impact disease development and outcome. Within the tumor microenvironment of GBM, tumor-associated microglia/macrophages (TAMs) play a large role in tumor development. Interestingly, TAMs in DMG display distinct features and have low immune activation in comparison to other pediatric gliomas. Although TAMs have been investigated substantially in GBM over the last years, this has not been the case for DMG due to the lack of tissue for research. Bit by bit, studies are exploring the TAM–glioma crosstalk to identify what factors within the DMG microenvironment play a role in the recruitment and polarization of TAMs. Although more research into the immune microenvironment is warranted, there is evidence that targeting or stimulating TAMs and their factors provide a potential treatment option for DMG. In this review, we provide insight into the current status of DMG research, assess the knowledge of the immune microenvironment in DMG and GBM, and present recent findings and therapeutic opportunities surrounding the TAM–glioma crosstalk.

## Introduction

Diffuse midline glioma (DMG), previously called diffuse intrinsic pontine glioma (DIPG), is a high-grade malignant pediatric tumor originating in the midline of the brain [1]. DMG is still uncurable and is the leading cause of pediatric brain tumor-related deaths [2]. The cause of DMG is unknown, although certain genetic conditions are known to predispose to brain gliomas [3]. The disease often occurs during middle childhood, with an incidence of 0.8 in 100,000 children per year at a median age of 6–7 years old in the United States [4]. Median survival post-diagnosis is less than 1 year, while approximately 10% of patients are still alive after 2 years [5].

The standard-of-care for newly diagnosed DMG patients is conventional fractionated radiation therapy, where a 54–60 Gy dose is delivered over 6 weeks [6, 7]. This provides only transient relief of symptoms in 70–80% of patients and a 3- to 4-month survival benefit [5, 8, 9]. Despite various clinical trials over the past few decades, no treatment has led to improvements in the prognosis of DMG for over 50 years. Predominantly the lack of biopsy and autopsy tissue resulted in a lack of knowledge. In clinical practice, treatment was therefore often guided by information from its adult counterpart, glioblastoma (GBM) [9]. GBM, a grade IV astrocytoma, is the most common malignant adult brain tumor, with an average incidence of 7.2 per 100,000 adults yearly [4, 10]. The median age of diagnosis is 64 and the median survival is just 15 months. In recent years, treatment by maximally safe surgical resection supplemented with chemotherapy and radiation has yielded moderate improvements [11]. Nowadays, significant molecular and cellular differences have been distinguished between both diseases, requiring separate research and treatment strategies [12].

Over the past few years, research on DMG has expanded considerably due to improvements in tissue acquisition and the generation of animal models, but also due to the discovery of unique mutations involved in DMG pathogenesis. In addition, current knowledge suggests that the tumor microenvironment is an important contributor to the DMG pathogenesis [13]. Particularly immune cells within the tumor microenvironment are presumed to play an important role in DMG, in analogy to reports in GBM [14, 15]. Early evidence suggests that DMG has a unique immune microenvironment with low T cell infiltration, presumably impacting prognosis, treatment options, and outcomes [12, 16]. Especially the presence, behavior, and activation state of tumor-associated microglia/macrophages (TAMs) in DMG warrant further research, as they have not received much attention in comparison to GBM [17, 18], despite their unique characteristics [19, 20]. The contribution of TAMs to disease progression has already been evaluated substantially in GBM over the past few years [18].

This review will present a brief overview of molecular and genomic characteristics of DMG and its immune microenvironment in comparison to GBM (Table 1). Furthermore, the contribution and potential therapeutic value of targeting TAMs in DMG are evaluated.

**Table 1:** Overview of the main clinicopathological features of DMG and GBM

	DMG	GBM
Median age of diagnosis	6–7 years old	64 years old
Survival	< 1 year	15 months
Tumor location	Midline of the brain	Throughout the brain; higher incidence in frontal and temporal lobes
Cell type of origin	Oligodendrocyte precursor cell	Neural stem cell/oligodendrocyte precursor cell
Frequent mutations	H3K27M, TP53, ACVR1, PDGFRA, PIK3R1/PIK3CA	TP53, PTEN, IDH1, NF1, EGFR, RB1
Tumor features	High genetic homogeneity, tissue histology grade II–IV	Low genetic homogeneity, heterogeneous tissue histology, often paired with a disrupted blood–brain barrier
Microenvironment features	Low levels of immune infiltration, TAM population is unpolarized	High levels of immunosuppression, TAM population resembles M0 phenotype
Treatment options	Radiation therapy	Surgical resection, radiation therapy, and chemotherapy

### Clinical presentation and diagnostics

The clinical presentation of DMG is led by the rapid development of symptoms 1–6 months before diagnosis [21]. These include clumsiness and difficulty in controlling eye movement, facial expression, and walking. Symptoms after clinical examination include the classic triad of cranial nerve palsies, long tract signs, and cerebellar deficits [22]. Signs of increased intracranial pressure can be present in case of expansion of the pons [21]. Magnetic resonance imaging (MRI) shows an infiltrative tumor centered around the pons, extending to the middle cerebellar peduncles, the midbrain, and the medulla [23].

Historically, stereotactic biopsies were routinely performed for histological diagnosis of DMG. These procedures carry the risk of surgical complications and are prone to sampling bias, as a biopsy from a single tumor locus may fail to capture intratumor heterogeneity [24]. Mainly due to the significant morbidity associated with biopsies and the implementation of MRI, a routine biopsy was discontinued in the 1990s [25]. While tumor resection aids diagnosis and can be curative for a subset of brain tumors, many tumors such as DMG are not amenable to (complete) surgical resection. This is due to the infiltrative nature and the high-risk neuroanatomical location of the tumor in the midline of the brain.

The development of rapid autopsy protocols for the collection of tumor tissue provided sufficient quantities of un-degraded DNA and RNA for molecular profiling and genome sequencing studies [26]. The analysis of autopsy specimens led to the discovery of new mutations such as H3K27M, ACVR1, PDGFRA, and PIK3R1/PIK3CA, which are presumed to play a role in the disease pathogenesis.

These mutations can now be diagnosed by analysis of cell-free, circulating tumor DNA (ctDNA) in cerebrospinal fluid or plasma/serum samples from peripheral blood of patients, so-called liquid biopsy [27]. Several studies successfully applied this technique in DMG and (pontine) GBM patients and were able to identify tumor-specific mutations in brain tumor-associated genes [28, 29]. Currently, most liquid biopsies utilize mutation-specific ctDNA assays, in which the remainder of the genome is unavailable for analysis [30]. Although liquid biopsies can therefore provide a good alternative for the diagnosis of established mutations, tumor tissue is likely to remain the standard for complete genomic profiling. However, novel approaches to liquid biopsy that survey greater parts of the genome are being explored [30].

### Biopsies for research purposes

Consequently, limited tissue has been available for research purposes. Autopsy protocols have been established to obtain live post-mortem DMG material to be used in translational models [31, 32]. While the generation of models derived from autopsy material allows for further research, these specimens have usually been exposed to radiotherapy and other treatments leading to genetic shifts, affecting the reliability of the model for untreated DMG. This has contributed to a poor understanding of the complex biology of DMG, based primarily on the evaluation of autopsy specimens or biopsies from patients with atypical MRI images [22]. In 2003, some institutions in Europe restarted performing relatively safe routine biopsies in children with suspected DMG, with no reported mortality and less than 10% of patients with transient reversible morbidities [33, 34]. This surgical success has led to an increase in available DMG tumor samples and contributed much to the current understanding of the disease. In the last five years, several research groups have successfully established patient-derived DMG cell lines from treatment-naïve patients. Although the amount of available DMG cell lines is increasing, the development of pre-clinical DMG models is still far behind that of supratentorial glioma models.

## Cellular and molecular features of DMG

### Origin and development

**Fig. 1:**
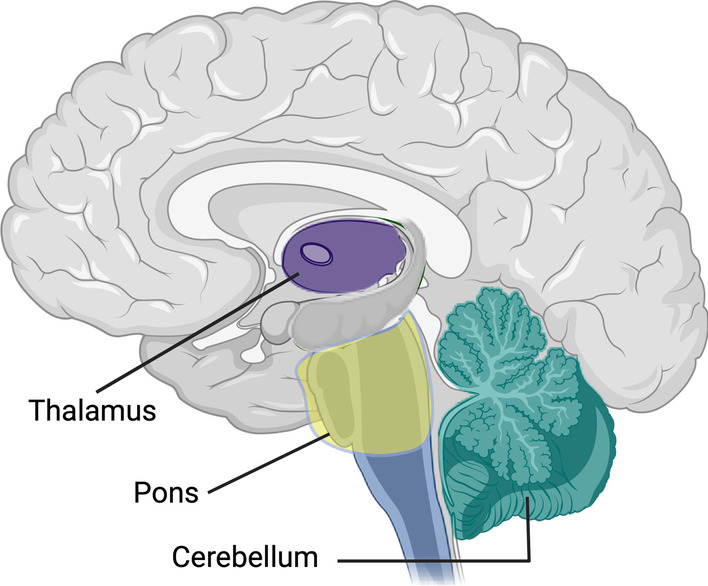
Overview of brain areas affected by the tumor in DMG patients. Most tumors are centered around the pons but can extend to the thalamus and cerebellum. Created with BioRender.com

DMG, as the name suggests, often arises in the midline of the brain, near the brainstem. The tumor is often centered around the pons, although it may extend to the thalamus and cerebellum (Fig. 1) [35]. Later in the course of the disease, subventricular spread of DMG can be present, contributing to morbidity and mortality [36].

In adult cancers such as GBM, a major obstacle for accurate diagnosis and comprehensive treatment is intratumor genetic heterogeneity. The presence of different molecular and phenotypical profiles within the same tumor specimen provides significant adaptability of the tumor to the environment [37]. As a result, biopsies may not be representative of the whole tumor and many potential drug targets are not homogeneously spread throughout the tumor tissue. This is associated with resistance to treatment and poor overall survival [38]. In contrast, in DMG the main driver mutations (such as H3K27M) and their partner mutations have been determined as spatially and temporally homogeneous [35]. This implies an early appearance of these mutations spreading throughout the brain during tumor evolution. The widespread presence of these mutations may provide useful treatment targets and make it easier to diagnose driver mutations by biopsy of a single tumor locus.

Monje et al. found that the peak age of onset of DMG during middle childhood is mirrored by the increased frequency of a spatially and temporally restricted neural precursor-like cell population [31]. During childhood, this cell type is restricted to the ventral pons and medulla, correlating with the area affected in high-grade gliomas. In addition, they found that this cell type expresses PDGFRA, suggesting sensitivity to PDGFRA gene amplifications, commonly observed in DMG. Histological staining at the sites of origin of DMG showed elevated levels of oligodendrocyte precursors and markers of oligodendrocyte precursors such as the intermediate filament proteins nestin and vimentin, and transcription factor olig2 are expressed in DMG cells [31]. Furthermore, single-cell RNA sequencing reveals that many oligodendroglial lineage as well as early neural precursor state genes are associated with DMG [39]. Therefore, the cell type of origin in DMG is most likely an oligodendrocyte precursor cell [31, 40]. For GBM, it is thought that there are three cells of origin: neural stem cells, neural stem cell-derived astrocytes, and oligodendrocyte precursor cells [41].

### Tissue characteristics

The majority of DMG tissue samples resemble other malignant gliomas in terms of vascular proliferation, tumor necrosis, mitotic activity, and anaplasia. Although there is homogeneity in driver mutations in different areas of the brain, there are differences in cell morphology between DMG patients. Histologically, DMG cases are classified as WHO grade II–IV tumors [42]. When examining pons samples, the study of Buczkowicz et al. reported predominantly GBM grade IV-like tissue based upon vascular endothelial proliferation and necrosis [24]. From the same patient, samples outside of the pons presented a histology consistent with anaplastic astrocytoma (AA), which is grade III instead. Although DMG, therefore, shows similarities to GBM and AA, it consists of heterogeneous tumor tissue (that expresses H3K27M), with characteristics of diffuse astrocytoma, AA, and GBM.

#### The blood–brain barrier (BBB)

The BBB is notorious for preventing a variety of cancer therapeutics from reaching the brain parenchyma. The integrity of the BBB is commonly detected by contrast-enhanced MRI using gadolinium-based contrast agents. Although cases vary, most DMG tumors show limited contrast-enhanced regions after administration of gadolinium, while other CNS tumors such as GBM show higher contrast levels within the brain [43, 44]. This suggests that the functioning of the BBB, just as in healthy brains, is preserved in DMG patients. The lack of improvement in DMG patients by therapeutically effective drugs such as temozolomide in GBM could therefore be the result of limited drug distribution across the intact BBB. In a PET assessment of zirconium-89 bevacizumab (a neutralizing antibody for VEGF-A) uptake in DMG patients, a positive, but not identical, correlation of MRI contrast enhancement and drug uptake was found [45]. Thus, the BBB might prevent the uptake of bevacizumab in DMG patients.

Additional evidence for a role of the BBB in DMG treatment failure was provided by in vitro assessment of chemosensitivity of primary DMG cultures. Direct exposure of primary DMG cells to a range of chemotherapeutics such as melphalan, doxorubicin, mitoxantrone, and carmustine revealed high levels of cytotoxicity [46, 47]. This indicates that DMG is sensitive to therapeutics, but that adequate delivery to the tumor is hindered. However, there are also contrasting findings that suggest that the unique features of the tumor or the drug result in treatment failure. In one DMG patient, the chemotherapeutic gemcitabine adequately reached DMG tissue, without impacting survival [48]. This could be due to inadequate drug concentrations or insufficient conversion to the active drug metabolite, or the result of uncharacterized biological factors. Other studies on panobinostat and palbociclib also indicate sufficient BBB and tumor penetration in DMG mouse models [49, 50]. The exact role of the BBB in DMG treatment failure is therefore not yet fully clear.

Nevertheless, an increasing number of research groups are exploring strategies to circumvent the BBB in drug delivery for diseases like DMG. One of the most promising methods is convection-enhanced delivery. Here, microcatheters are placed in the tumor. Several reports have shown high safety and feasibility of adequate drug delivery using this method [51, 52]. A less invasive approach is focused ultrasound-mediated BBB opening, which is capable of increasing the effectiveness of systemic drug delivery in a variety of brain pathologies [53, 54].

### Genomic alterations

While histologic features between pediatric and adult high-grade gliomas are similar, DMG has a unique molecular landscape. Recurring mutations such as H3K27M, TP53, ACVR1, PDGFRA and PIK3CA are linked to key oncogenic pathways and may present therapeutic targets [55, 56]. Particularly histone H3 mutations, which are very infrequently found in adult high-grade gliomas, are highly present in DMG patients [57, 58]. Looking at the genes commonly mutated in GBM, these include TP53, PTEN, IDH1, NF1, EGFR, and RB1 [59].

**Fig. 2:**
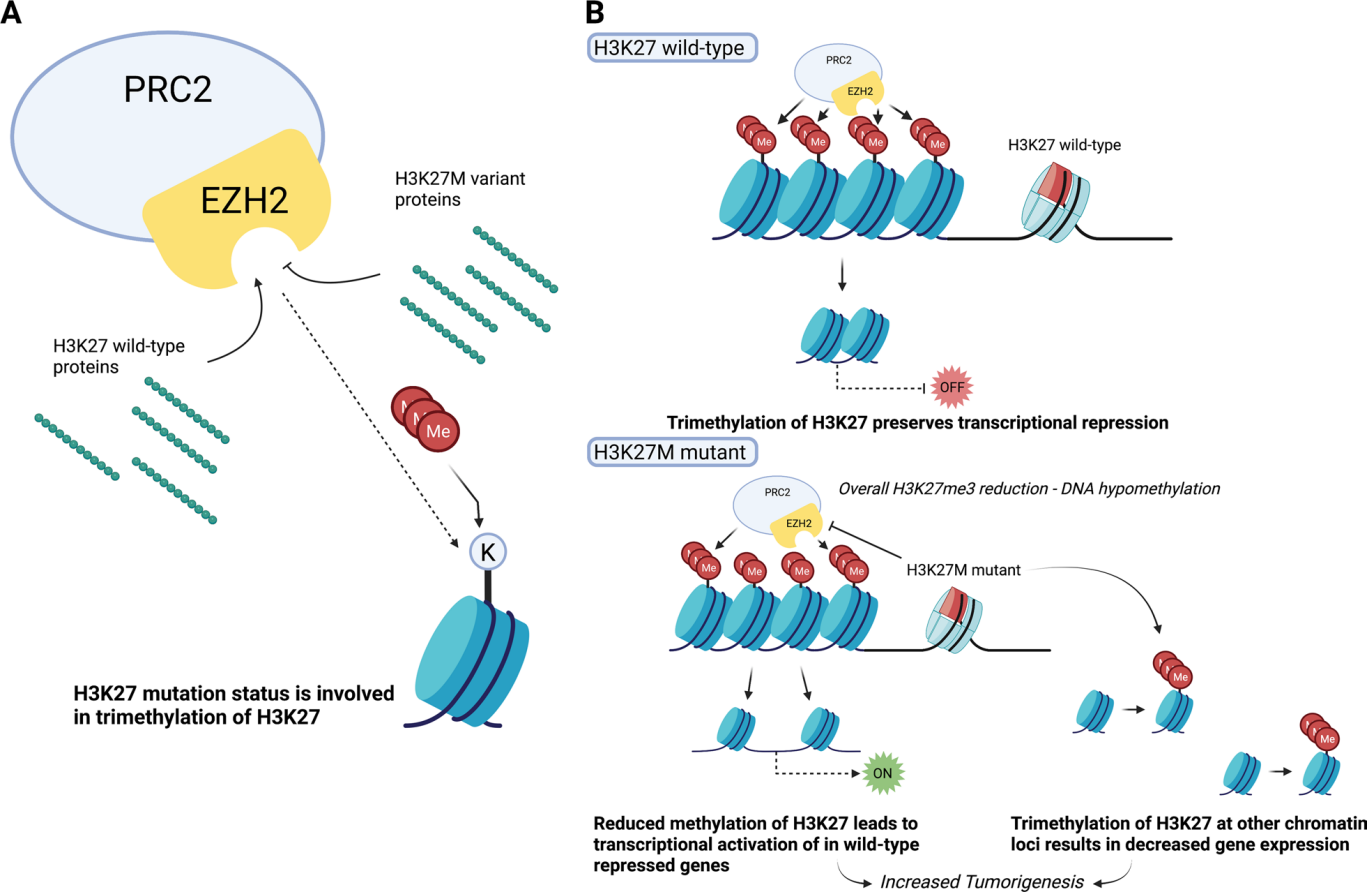
The H3K27M variant interferes with the EZH2 subunit within the PRC2 complex.** A** The EZH2 subunit within the PRC2 complex is involved in histone methylation and subsequent transcriptional repression. H3K27M variant proteins interfere with histone methylation through an inhibitory interaction with the EZH2 subunit, while wild-type proteins do not. **B** In the H3K27 wild type, trimethylation of lysine residues preserves transcriptional repression. In the H3K27M variant, methylation is reduced, resulting in chromatin unwinding and increased gene expression of genes repressed in the H3K27 wild type. Additionally, a focal gain of methylation at other loci results in decreased gene expression elsewhere. Created with BioRender.com

**Fig. 3:**
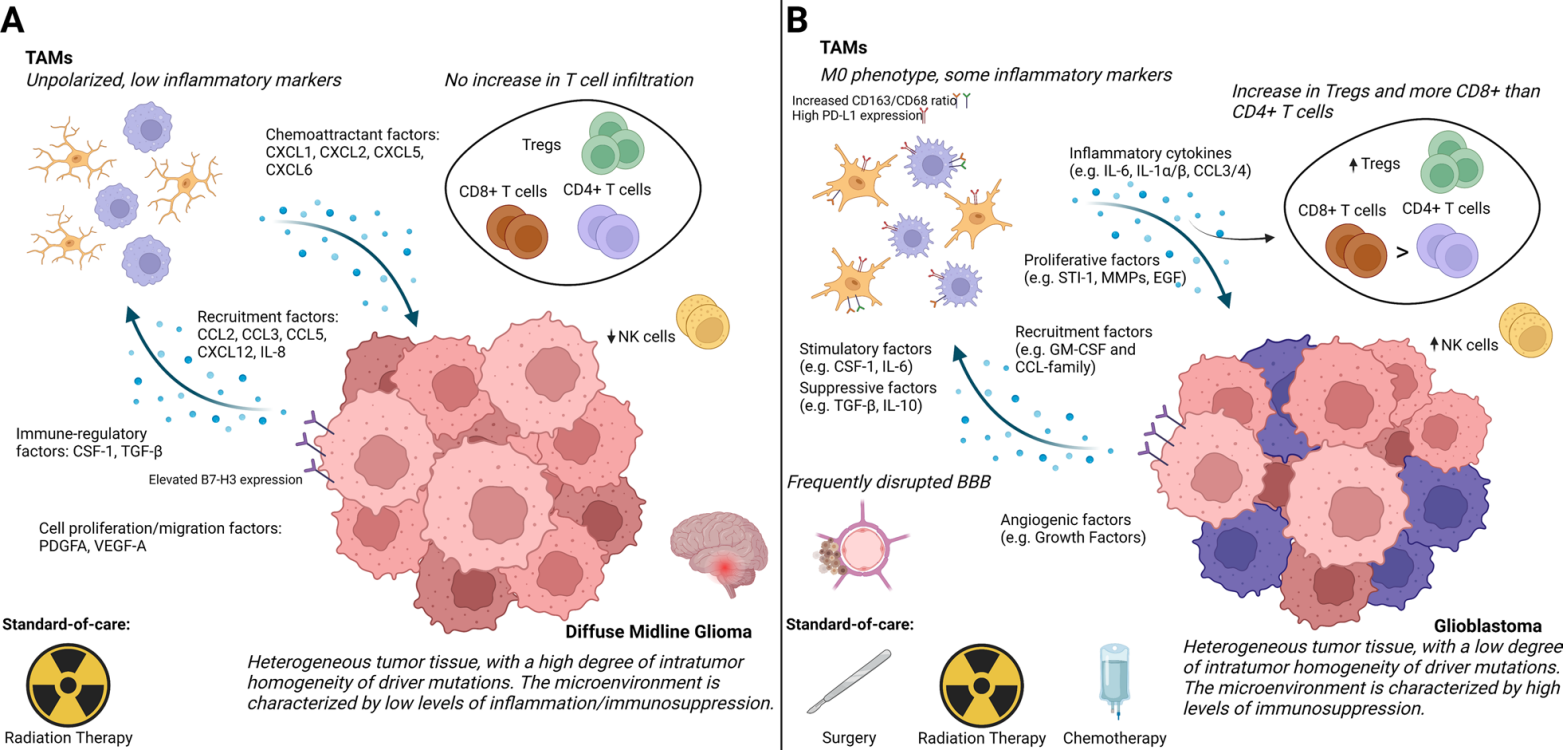
Comparison of the immune microenvironment in DMG and GBM.** A** DMG is found in the midline of the brain and consists of heterogeneous tumor tissue with a high spatial and temporal homogeneity in driver mutations. The microenvironment shows low levels of immune infiltration, inflammation, and immunosuppression. There is no increase in T cell infiltration compared to other pediatric gliomas, while there is a decrease in natural killer (NK) cells compared to healthy children. The TAM population (consisting of microglia and macrophages) is unpolarized, expressing low levels of pro-inflammatory markers. Only selected cytokine/chemokine factors have been confirmed in DMG. The standard-of-care for DMG is radiation therapy. **B** GBM is found throughout the brain, consists of heterogeneous tumor tissue with low spatial and temporal homogeneity of driver mutations, and often coincides with a disrupted blood–brain barrier (BBB). The microenvironment shows high levels of immunosuppression, characterized by infiltration of Treg T cells. In addition, the ratio of CD8 + to CD4 + T cells and the number of NK cells in the tumor are increased. The TAM population in GBM shows some pro-inflammatory markers and is associated with an M0 phenotype. TAMs show an increase in the CD163/CD68 ratio, with increased PD-L1 expression. Many cytokines and chemokines have been found in GBM samples, of which a selection is shown in the figure. The standard-of-care for GBM is surgery, radiation therapy, and chemotherapy. Created with BioRender.com

Nearly 50% of all pediatric high-grade gliomas (pHGGs) carry mutations in the genes coding for histone variants H3.1 and H3.3, with high-grade midline gliomas between 60 and 80% [57]. Most prevalent is the lysine 27 to methionine (K27M) mutation in histone H3. The H3K27M variant leads to aberrant recruitment of the multiprotein polycomb repressive complex 2 (PRC2) and enzymatic inhibition of the histone-lysine N-methyltransferase Enhancer of zeste homolog 2 (EZH2) (Fig. 3) [60, 61]. This complex plays a role in the maintenance of transcriptional silencing through trimethylation of K27 of histone H3 (H3K27me3) (Fig. 2). Because of the K27M mutation, K27 is hypomethylated, which promotes a more accessible chromatin state, followed by decreased transcriptional repression. This increased transcription is likely to contribute to DMG oncogenesis [60, 62]. The K27M variant, therefore, contributes to a global reduction of H3K27me3, but paradoxically also leads to a methylation increase in genes associated with various cancer pathways. This might be due to the recruitment or retention of the EZH2 subunit in presence of the mutant K27M protein at these loci [63]. Together, this has been proposed to reprogram the epigenetic landscape and drive DMG tumorigenesis.

The presence of the H3K27M mutation is associated with a significant decrease (by 2.3 years) in overall survival as compared to other pHGGs [64]. Recent meta-analyses have linked the presence of the K27M mutation in specific histone variants to unique survival statistics. Mackay et al. demonstrated that K27M mutations in H3.1 and H3.3 relate to worse survival prognoses and are generally present in younger patients [16]. Additionally, the H3.1 K27M and H3.3 K27M mutations seem to be distinct in their oncogenic programs and prognosis, with the latter linked to the worst response to treatment [65].

Because of the recurring presence and contribution of histone 3 mutations on the clinical course of the disease, these tumors are classified by the WHO as grade IV tumors since 2016, even in absence of concurrent histology [1]. Around 80% of DIPG cases harbor these mutations and these have been reclassified as ‘Diffuse Midline Glioma, H3 K27M-mutant’ [57].

## The tumor microenvironment in DMG and GBM

Apart from tumor-intrinsic differences, a growing body of evidence suggests that the cellular tumor microenvironment plays a significant role in tumor formation, progression, and treatment [15, 66]. This microenvironment consists of a variety of stromal cells, including non-neoplastic cell types such as fibroblasts, immune cells, and epithelial cells.

The first evidence for a possible role of the microenvironment in DMG dates back to 2014, from a study in which post-mortem tumor material was used in the development of xenograft mouse models. This material was used either directly, to preserve as much as possible of the original tumor microenvironment or indirectly after in vitro expansion. Surprisingly, orthotopically xenografting post-mortem tumor material directly into the pons of immunodeficient mice resulted in tumors composed of murine cells, while the xenografts using tissue which was cultured first resulted in tumors composed of human cells [67]. The purpose of the direct method was to avoid loss of growth factors present in the tumor microenvironment after in vitro culture, while the indirect method allowed for cell expansion. The development of a murine tumor instead of a human tumor after direct xenotransplantation is observed very infrequently, and it is presumed to be the result of cell–cell fusion [68, 69]. Additionally, studies have demonstrated that in cell–cell fusion, DNA of both species is present and proteins of both species are expressed [70, 71]. In the study by Caretti et al., xenotransplantation resulted, in all cases, in murine tumors, without evidence of the presence of human DNA. However, alternative strategies such as whole genome sequencing or analysis of the xenograft directly after injection are needed to confirm this result. Although it remains unclear whether the generation of murine tumor cells might be due to the transplanted human microenvironment or to the fact that the mice are immunodeficient, this work highlights the importance of careful consideration of the tumor and its environment in DMG, especially in the development of such translational models.

Particularly immune cells in the tumor microenvironment have the potential to affect the tumor through the production of growth and survival factors, cytokines, and chemokines. In the tumor-specific immune response, CD8 + (cytotoxic) T cells and Treg (regulatory) T cells play a central role. CD8 + T cells represent the combat arm of the tumor-specific immune response and are more prevalent in GBM than CD4 + T helper cells [72]. An increase in CD8 + T cell infiltration has been linked to better survival outcomes in various studies. On the other hand, Treg T cells inhibit the CD8 + response. Although debated, some studies suggest that increased numbers of Treg T cells are linked to a worse outcome in GBM [73]. For DMG, this is not known.

The role of immune cells in the tumorigenesis of GBM has been explored in recent years. Several studies have shown that neoplastic cell growth and development are stimulated by changes in the local immune environment [14, 74]. Up until recently, the immune microenvironment for DMG has not been characterized. One study made direct comparisons of adult GBM and DMG tissue samples and found differences in leukocyte composition and the levels of cytokines/chemokines, suggesting a low inflammatory signature of the immune microenvironment in DMG compared to GBM [20]. In addition, another publication suggests the presence of a unique non-inflammatory immune microenvironment in DMG [19].

### ‘Immune cold’ state

The inflammatory state of gliomas is often assessed by T cell infiltration. GBM tumors are considered ‘immunologically cold’, as the levels of T cell infiltration are significantly lower compared to other high-grade gliomas. Looking at DMG, the study of Lin et al. found that the fraction of T lymphocytes (CD3 +) of total infiltrated leukocytes (CD45 +) is strikingly lower in DMG samples compared to GBM. Whereas T lymphocytes comprise about 7–50% of total leukocytes in GBM tissue, this is only around 2% for DMG. This was the case for both post-mortem samples and pre-treatment biopsy samples, described in this study. The low levels of lymphocyte infiltration were confirmed by two other recent studies [19, 75]. Additionally, Lieberman et al. found that both pediatric low-grade glioma (pLGG) and pHGG had increased infiltration of T cells, particularly CD8 + T cells, over different pediatric non-tumor tissue. The numbers of T cells were not increased over control for DMG [19].

In GBM, the immunosuppressive mechanisms are well-characterized and likely play a large role in the pathogenesis [76]. In pHGG, there is a significantly elevated concentration of immunosuppressive factor programmed death ligand 1 (PD-L1), and factors B7-H3 and transforming growth factor-beta 1 (TGF-ß1) show increased mRNA expression. Only B7-H3, vascular endothelial growth factor A (VEGF)-A, and platelet-derived growth factor subunit A (PDGFA) have increased expression in DMG [19, 20]. Glioma cell secretome analysis by Lin et al. measured DMG or GBM-derived factors and found that DMG and pediatric GBM cells secrete fewer cytokines and chemokines than adult GBM cells. Congruently, patient-derived DMG cell cultures expressed no cytokine genes and only a few chemokines and growth factors [20]. Noteworthy is the absence of cytokines in DMG cultures for the recruitment of immune cells to the tumor microenvironment, confirmed by Lieberman et al. [19]. Together, these findings provide evidence for much lower levels of immunosuppressive factors and general immunosurveillance in DMG, as opposed to pHGG and GBM.

NK cells are another cytotoxic type of lymphocyte that plays a role in the innate immune system. In brain tumors such as GBM, the anti-tumor activity of NK cells is often suppressed [76]. Nevertheless, analysis of GBM tumor samples showed increased infiltration of NK cells as opposed to pilocytic astrocytoma [77]. On the contrary, lymphocyte profile testing using blood samples showed a decreased number of NK cells in DMG patients as compared to healthy control subjects [78]. Not much is known about the contribution of NK cells to the pathogenesis of DMG. In vitro, NK cells can lyse DMG cells, possibly mediated by expression of NKG2D ligands by DMG cells [19].

DMG tumors are therefore considered to be in an ‘immune cold’ state just like GBM, but probably to a much higher degree. The balance of lymphocyte levels and especially combatting immunosuppression has been and still is a promising therapeutic approach in GBM [76]. In DMG, the lack of infiltrating lymphocytes suggests that these do not play a central role in the early or late pathogenesis. The immune infiltrate has been linked to survival in GBM [79], but in pediatric brain tumors it does not seem to correlate with survival or mutational load [80]. In addition, the much lower degree of mutational load (which predicts worse response rates to immunotherapies) in pediatric tumors may be a reason for the absence of a correlation with the immune infiltrate [81].

However, early evidence suggests that while DMG shows low lymphocyte infiltration, the tumor cannot be completely regarded ‘immune cold’. Another study found low T cell infiltration in DMG and hemispheric pHGG and corroborated findings of previous studies [20, 75]. Further comparison of gene expression profiles of DMG and hemispheric pHGG found that DMGs had higher expression of genes involved in chemokine and cytokine signaling, leukocyte, and macrophage functioning [75]. This indicates that although there is minimal T cell infiltration, there is significant immune signaling in DMG and it may not be as immunologically inactive as assumed historically.

### Infiltration of myeloid cells

Microglia, the resident immune cells of the central nervous system (CNS), as well as bone marrow-derived macrophages (BMDMs) are potent immune cells and around 30–50% of cells in low and high-grade glioma are immune cells [82, 83]. The role of microglia and macrophages within the immune microenvironment of DMG has only recently been studied. Conventionally, microglia/macrophages are marked by the expression of CD11b, whereas an additional high CD45 expression distinguishes macrophages from microglia (low CD45) [84]. The robustness of this categorization is debatable, as microglia upregulate CD45 in response to inflammation [85, 86]. Novel microglia-specific markers and other strategies of determining the proportion of microglia and macrophages in GBM have been explored, but have not proven to be conclusive [82, 87, 88]. Up to date, it is not possible to unambiguously discriminate between resident microglia and (peripheral) macrophages that invaded the brain parenchyma, although vast efforts have been made [89]. Microglia- or macrophage-like cells in the brain are therefore often referred to as “microglia/macrophages”.

One of the first reports evaluating microglia/macrophages in DMG tissue mentions the presence of a significant number of microglia/macrophages as opposed to lymphocytes in post-mortem tissue samples (Caretti et al., 2014). A more recent study found that when looking at the proportion of CD11b + myeloid cells in the CD45 + leukocyte population, this percentage was significantly higher in DMG samples compared to adult GBM (95 vs. 70%) [20]. This indicates that in GBM, non-myeloid immune cells are present. The high levels of CD45 + /CD11b + cells indicate a significant myeloid-only immune cell infiltration in DMG, in line with the study of Caretti et al. in 2014. However, the study by Lin et al. also analyzed healthy pediatric cortical tissue and report 97% CD11b + cells of CD45 + leukocytes [20]. Although DMG differs from GBM in the proportion of CD11b + myeloid cells, this percentage in DMG is similar to healthy tissue in the same developmental timeframe of the brain.

The study by Lieberman et al. also measured myeloid cell infiltration by using other markers. Microglia and macrophages both express general marker CD68, as well as CD163, a marker for their alternatively activated or anti-inflammatory state. The proportion of CD68 + cells that also shows CD163 positivity is often used as a measure of immunosuppression. An initial comparison of the number of CD68 + myeloid cells found no differences in immune infiltration between DMG, pHGG, or pLGG [19]. However, DMG samples showed no increase in CD163 + cells, whereas pHGG and pLGG did. Consequentially, pHGG and pLGG had significantly elevated CD163 + /CD68 + ratios of macrophages compared to control, suggesting a degree of immunosuppression in these diseases, but not in DMG. This suggests that while total myeloid infiltration in DMG and other pediatric gliomas is similar, myeloid cells in DMG do not display an immunosuppressive phenotype.

### Activation state

Macrophages have classically been divided into two activation states, M1 and M2, although this classification is highly debated. An M1 phenotype represents an anti-tumor response, while M2 points towards pro-tumorigenic behavior [90]. Using defined gene sets for these states, transcriptome profiling allows for the classification of the activation state [91]. In GBM, the high degree of immunosuppression has been partly attributed to the accumulation of M2-polarized cells [92, 93]. However, a recent more comprehensive phenotypic and genotypic characterization of GBM-infiltrated innate immune cells revealed a distinct M1–M2 continuum, closer to an undifferentiated or nonpolarized M0 phenotype for GBM-associated myeloid cells [94]. Among other differences, Gabrusiewicz et al. demonstrated that these GBM-associated myeloid cells express lower levels of CD163, TGF-ß1, interleukin (IL)-1ß, and TNF-α, compared to M2- and M1-polarized macrophages [95].

Studies of the morphology and expression profiles of infiltrative myeloid cells in DMG showed significant differences compared to pLGG and pHGG. In the study by Lin et al., DMG-associated macrophages did not fit properly into the M1/M2 phenotype. Pre-ranked gene-set enrichment analysis on significant differentially expressed genes between DMG-associated macrophages and normal cortical microglia indicated no enrichment for M1 or M2 defined gene sets [20, 91]. However, gene ontology analysis revealed that these DMG-associated macrophages do exhibit some degree of activation, as terms such as ‘response to hypoxia’ and ‘antigen processing and presentation’ were upregulated as compared to cortical microglia. Furthermore, of the gene sets expressed, DMG samples were significantly less inflammatory as compared to GBM samples. Top genes upregulated for DMG were related to cell adhesion, angiogenesis, and extracellular matrix organization. In contrast, GBM terms include many inflammation-associated terms related to monocyte chemotaxis, neutrophil chemotaxis, and the chemokine-mediated signaling pathway. Macrophages within the immune microenvironment in DMG and GBM, therefore, exhibit distinct gene expression profiles and activation states.

Another method to assess macrophage polarization resulted in the coculturing of macrophages with three patient-derived DIPG cultures (two with an H3.3 K27M mutation and one with an H3.1 K27M mutation) or with the adult GBM U87 cell line, which is a known positive control for immunosuppression [19]. The H3.1 K27M DMG culture and the U87 cell line produced VEGF and IL-6 at high levels, along with a small amount of IL-10. These cytokines have been implicated in the immunosuppressive polarization of macrophages. The H3.3 K27M DMG cultures had lower VEGF levels and no detectable IL-6 or IL-10 levels. In addition, the U87 cell line and H3.1 DMG culture produced macrophage colony-stimulating factor (CSF) along with chemokines that attract circulating monocytes, suggesting that monocytes are attracted to tumors predisposed to repolarize monocytes. This was confirmed following coculture with healthy donor monocytes with an unpolarized M0 phenotype. The U87 culture induced the upregulation of immunosuppression markers such as CD163 and PD-L1 in macrophages. In the H3.1 DMG culture, the upregulation of immunosuppression markers was somewhat smaller as compared to the U87 culture. The H3.3 DMG cultures only had a small effect on the macrophage phenotype. Together these findings confirm that DMG cultures are not likely to induce an immunosuppressive phenotype in macrophages [20].

Interestingly, the total lack of monocyte recruitment factors in some DMG (H3.3) cultures makes it unclear how macrophages are differentially repolarized, as seen in the study by Lin et al. However, due to the paucity of such studies, future research is needed to confirm crosstalk between DMG cells and macrophages and in what sense this contributes to a tumorigenic phenotype.

## Glioma–TAM crosstalk

Numerous studies revealed that TAMs play a large role in tumorigenesis and glioma progression. Although the mechanisms by which TAMs affect glioma cells may differ from tumor to tumor, several potent tumor-affecting factors have been identified. In contrast to adult high-grade glioma, TAMs exhibit anti-tumoral properties in medulloblastoma, a common malignant pediatric brain tumor [96]. In GBM, considerable progress has been made in elucidating the relation between specific glioma factors to the contribution of TAMs in the immune microenvironment to promote tumorigenesis. A variety of factors known from involvement in GBM are discussed below. It is unclear whether these are involved in DMG pathophysiology, as much more research on the microenvironment in DMG is needed to confirm potential etiologies.

### Factors recruiting TAMs

Lin et al. showed that DMGs express C-C motif ligand (CCL) 2 and CCL5, but the levels were not different compared to pLGG or pHGG according to Lieberman et al. [19, 20]. CCL2, alternatively named monocyte chemoattractant protein (MCP)-1, is an inflammatory chemokine that is essential for the recruitment of macrophages and Tregs T cells, leading to the immunosuppression seen in GBM [38, 97]. CCL2 is expressed by a variety of astrocytoma and GBM cell lines in vitro and in vivo [98, 99]. Human GBM tumor samples in vitro displayed a positive correlation between CCL2 levels and infiltration of TAMs [100]. The level of CCL2 expression is linked to glioma aggressiveness in a CCL2-expressing CNS-1 glioma animal model [101]. Stimulation of the CCL2 receptor, C-C motif receptor (CCR) 2, on microglia resulted in an increase in glioma invasiveness, possibly regulated by microglial IL-6 production [102]. However, the role of CCL2 has been disputed, as many TAMs infiltrate independently of CCR2 signaling and CCL7 is observed to have a stronger relation to TAM infiltration [103, 104].

CCL5 is, along with CCL2, a highly expressed versatile inflammatory mediator that attracts macrophages, T cells, and granulocytes in multiple types of cancer [105, 106]. In GBM, CCL5 can be expressed by the glioma or stromal cells and directly promote proliferation and migration of microglia/macrophages, but can also indirectly alter the microenvironment by recruiting inflammatory effector cells [107, 108]. A recent study on microglia–glioma interactions links blockage of its receptor, CCR5, to the prevention of conversion to the M2 phenotype, along with reduced overall microglia migration in presence of glioma-secreted factors [109].

Another recruiting factor elevated in DMG samples is the C-X-C motif ligand (CXCL) 12, also known as stromal-derived factor 1 (SDF-1), which also promotes recruitment of TAMs in HGGs. The CXCL12/CXCR4 axis has been implicated in GBM resistance to irradiation [110]. In a murine astrocytoma model, this chemokine is especially potent in attracting TAMs to hypoxic areas in the brain [111]. In vitro, stimulation with CXCL12 elevated cell invasion by DMG cell lines in invasion assays [112]. More research is needed to confirm the overall presence of these chemoattractants and their subsequent relation with DMG pathophysiology.

Another highly expressed chemokine in DMG samples is IL-8 (CXCL8) [19, 20, 75]. Expression of IL-8, also known as neutrophil chemotactic factor, has been linked to cell proliferation, invasion, and vascular mimicry (a tumor cell-driven form of neovascularization) in GBM [113]. IL-8 (and CXCL2) can induce CXCR2 signaling, which is linked to the vascular mimicry observed in GBM. The IL-8/CXCR2 axis is stimulated after treatment with anti-angiogenic therapies, resulting in more frequent GBM relapses and eventual resistance to anti-angiogenic treatment [114, 115]. Treatment with CXCR2 antagonist SB225002 resulted in a decrease in glioma growth by preventing vascular mimicry in mice models [116, 117]. However, as anti-angiogenic therapies provide no survival benefit in DMG, further research is needed to assess the role of the IL-8, the IL-8/CXCR2 axis, and its therapeutic potential in DMG.

The CSF family of cytokines has been linked to macrophage infiltration and activation in GBM and DMG [20]. Granulocyte–macrophage CSF (GM-CSF) drives microglia-dependent glioma invasion in vitro, while mRNA expression of its gene CSF-2 has been inversely associated with patient survival in GBM [118]. Furthermore, CSF-1 is secreted by glioma cells and inhibition of its receptor reduced the density of TAMs and attenuated GBM invasion in vivo [119]. CSF-1 is also able to steer TAMs towards the pro-tumorigenic M2 phenotype [120].

### Pro-tumorigenic effects of TAMs

TAMs produce a variety of paracrine factors that support glioma growth [121]. The co-chaperone stress-inducible protein 1 (STI-1) is such a factor. There is evidence for a link between the expression of STI-1 and glioma cell behavior, as high STI-1 expression promoted proliferation in the U87MG cell line as well as migration in GBM95 cell line in vitro [122]. In a GL261 glioma mouse model, STI-1 expression increased the proliferation of GBM cells in vivo, but the receptors involved are not fully characterized yet [123].

TGF-ß is implicated in early and late oncogenesis, due to its effects on cell proliferation, tumor invasion, and angiogenesis [124]. TAMs are a major source of TGF-ß [125]. Disruption of TGF-ß-induced signal transduction leads to a decrease in GBM invasiveness, migration, and tumorigenicity in mice [126]. Additionally, TGF-ß2 induces the expression of matrix metalloprotease (MMP) 2 and suppresses the expression of tissue inhibitor of metalloproteinases (TIMP) 2, destroying the extracellular matrix [127]. For DMG, there is evidence that TGF-ß2 gene expression is higher than in normal tissue and low-grade glioma and similar to pediatric GBM [128]. Also, DMG tumor cell cultures have been shown to secrete similar levels of TGF-ß as the U87 cell line [19]. Systemic inhibition of TGF signaling is being considered as a potential treatment strategy, but clinical trials have so far not yielded consistently encouraging results.

Recently, a correlation between high levels of platelet-derived growth factor-beta (PDGFβ) and a high presence of IBA1 + TAMs in DMG and hemispheric pediatric glioma was observed. Human DMG and hemispheric brain tumors with high PDGFß are characterized by a stronger inflammatory response [75]. Interestingly, BMDMs upregulate chemokine expression CCL2, CCL7, and CCL12 when stimulated with PDGFß, while cultured microglia do not. While it is not yet possible to discriminate between BMDMs and microglia as mentioned before, these results could indicate the need for cell-type-specific treatment of the total TAM population. By generating PDGFß-driven tumors followed by genetic ablation of individual chemokines, Ross et al. provided evidence for survival benefit in animal models when CCL3 was knocked out. CCL3 binds to CCR1 and CCR4 to induce chemoattraction of neutrophils, monocytes, T cells, B cells, and dendritic cells [129, 130]. Together, these findings provide evidence for a role of PDGFß and CCL3 in the recruitment of TAMs, although further investigation is needed. An overview of the molecules associated with DMG can be found in Table 2.

**Table 2:** Overview of molecules associated with the immune microenvironment of DMG

Factor type	Factor	Findings	Reference
Chemoattractant or inflammatory	CXCL1, CXCL2, CXCL5, CXCL6	Human DMG tissue samples showed significant gene expression of the CXCR2 pathway, which includes CXCL1, CXCL2, CXCL5, and CXCL6 as compared to other human pHGGs	[12]
Recruitment	CCL2, CCL5, CXCL12	Analysis of single-cell DMG gene expression and secreted protein levels from biopsy tissue included CCL2, CCL5, and CXCL12	[20]
	CCL3, PDGFß	High PDGFß levels in human DMG tissue samples are linked to an increased presence of IBA1 + TAMs. In a murine PDGFß-driven tumor model, CCL3 knock-out provided survival benefit	[75]
	IL-8	Ross et al. found increased IL-8 gene expression in human DMG tissue samples. Lieberman et al. found that IL-8 was significantly increased over normal and pLGG in surgery and autopsy DMG tissue, but lower than pHGG	[12, 19]
Immune regulatory	CSF-1, TGF-ß	Analysis of single-cell DMG gene expression and secreted protein levels from biopsy tissue included CSF-1 and TGF-ß	[20]
Cell proliferation or migration	PDGFA	Analysis of single-cell DMG gene expression and secreted protein levels from biopsy tissue included PDGFA	[20]
	B7-H3, VEGF-A	Within the tumor microenvironment of surgery and autopsy DMG tissue, factors associated with immunosuppression B7-H3 and VEGF-A were increased relative to control	[19]

C-X-C motif ligand (CXCL); diffuse midline glioma (DMG); C-X-C chemokine receptor (CXCR); C-C motif ligand (CCL); platelet-derived growth factor-beta (PDGFß); ionized calcium binding adaptor molecule 1 (IBA1 +); interleukin-8 (IL-8); pediatric low-grade glioma (pLGG); pediatric high-grade glioma (pHGG); colony-stimulating factor 1 (CSF-1); transforming growth factor-beta (TGF-ß); platelet-derived growth factor subunit A (PDGFA); B7 homolog 3 protein (B7-H3); vascular endothelial growth factor A (VEGF-A). For GBM, an overview of the key molecules associated with the microenvironment has been published recently [131].

### Potential contribution of TAMs in DMG treatment

In cancer, the potential therapeutic application of TAMs is gaining attention [18, 132]. In various clinical trials the depletion, attraction, or reprogramming of macrophages in solid and blood cancers is targeted, but only a few of these approaches are specifically tested in GBM [17]. In DMG, a handful of immunotherapeutic targets has been explored and only a few target TAMs or their factors directly. Below, we highlight some recent studies that (in)directly involve TAMs to treat DMG.

VEGF-A is among the factors with elevated levels in DMG samples that can be secreted by microglia [19, 133]. VEGF is a frequently studied drug target of the tumor microenvironment in gliomas, as it is involved in promoting angiogenesis and vascularization [134, 135]. A high expression of VEGF-A and/or its receptor VEGFR2 in cancer has been associated with poor prognosis in various clinical studies [136]. Bevacizumab is a recombinant humanized monoclonal antibody that selectively binds and neutralizes all isoforms of VEGF-A [134]. Bevacizumab has been approved for use in recurrent GBM as it demonstrated significant progression-free survival rates as a single agent [137]. Clinical studies fail to demonstrate an increase in overall survival, which is why it is not approved for newly diagnosed GBM patients [137, 138]. In DMG patients, bevacizumab treatment had minimal efficacy [139]. The lack of effect was evaluated in DMG mouse models and was due to the presence of an intact BBB, which coincided with low VEGF expression and the inability of bevacizumab to target the tumor [140]. For this reason, methods of drug delivery across the BBB are being explored to use drugs such as bevacizumab as a treatment for DMG [141].

Another potential treatment for DMG targets the anti-phagocytic CD47 and signal regulatory protein alpha (SIRPα) interaction. Glioma cells are known to express a combination of pro-phagocytic (eat me) and anti-phagocytic (don’t eat me) signals. CD47 has been identified as the primary and crucial ‘don’t eat me’ signal on the cell surface of many cancers [142, 143]. CD47 binds and activates SIRPα, an inhibitory protein on the surface of myeloid cells, which subsequently initiates a signaling cascade that inhibits phagocytosis by myeloid cells. By using anti-CD47 antibodies, phagocytosis by myeloid cells is promoted [144]. Most non-neoplastic cells have little to no CD47 expression and are not affected by CD47-blocking antibodies. Targeting the CD47-SIRPα interaction may therefore selectively treat gliomas such as GBM and DMG. Treatment with a mouse anti-human CD47 monoclonal antibody demonstrated therapeutic efficacy in patient-derived orthotopic xenograft mouse models [145]. Macrophage-mediated phagocytosis of tumor cells was significantly elevated in vitro, while tumor growth and tumor burden were significantly reduced in xenograft mouse models. There have been no clinical trials for anti-CD47 treatment in pHGG yet. However, a phase I trial did show high tolerability in adults with various (non-CNS) solid cancers [146]. The complex nature of TAMs in the CNS warrants more research. Moving TAMs towards a phagocytic phenotype and thereby preventing or altering a pro-tumorigenic state could be particularly valuable in DMG. However, more research is needed to confirm the penetration of the BBB and subsequent interaction with the tumor.

The discovery that the H3K27M mutation via EZH2 profoundly influences the DMG transcriptome and biology led to an interest in targeting this enzyme [147]. Further inhibition of the EZH2 enzyme is thought to decrease trimethylation more, resulting in downregulation of genes associated with cancer pathways. Several inhibitors to target EZH2 gene expression have been developed. In DMG preclinical mouse models, EZH2 inhibition leads to a reduction in tumor growth and extended survival [147]. To investigate tumoral effects, antisense RNA strategies were used to target EZH2 gene expression in microglia and glioma cells [148]. In vitro, only extended treatment (to one week) of DMG H3K27M cells with EZH2 inhibitors had significant effects on the proliferative and survival capabilities of glioma cells (independent of mutational status). However, treatment of microglia directly led to a decrease in H3K27me3 levels and resulted in anti-tumoral properties such as increased phagocytosis and IL-1β secretion. In a coculture of microglia with either wild type or H3K27M-mutant type cells, microglia treated with antisense RNA led to a decrease in glioma cell transwell Matrigel invasion while phagocytosis and glioma cell-death were increased.

## Concluding remarks

DMG remains an almost universally fatal disease, for which there is no effective treatment yet. Currently, the tumor microenvironment in DMG has been insufficiently studied. Due to the lack of patient material and the modest similarities to its adult counterpart, clinical care of DMG has long been based upon experience with GBM. However, recent research presents unique characteristics in DMG regarding mutations and origin, but also in terms of lymphocyte infiltration, expression of immune-modulatory factors, and myeloid behavior as compared to GBM (Fig. 3). Within the tumor microenvironment, TAMs in GBM and DMG show distinct activation states. The influence of TAMs in the progression of GBM has been studied substantially and there is evidence for a role of TAMs in disease progression. In DMG, there is a possible role of microglia, macrophages, and their secreted factors in its pathophysiology as well.

The precise functioning and contribution of TAMs in combination with the ‘immune cold’ state of DMG is not known yet. Early clinical research using TAM-targeted interventions show anti-tumoral effects in vitro. Nevertheless, knowledge of the immune microenvironment is limited and more research is needed to fully characterize its nature to develop immunotherapies that comprehensively target the whole tumor. Moreover, the BBB seems to be a major hurdle in the delivery of drugs to the tumor. Strategies to open the BBB will benefit the development of novel large-molecule drugs in DMG, but also other brain tumors. Future research endeavors on further characterization of the tumor microenvironment and the TAM–glioma crosstalk could provide promising targets for the development of novel immunotherapies in the treatment of DMG.

## Data Availability

Not applicable.

## Abbreviations

AA: Anaplastic astrocytoma
BBB: Blood–brain barrier
BMDM: Bone marrow-derived macrophage
CCL: C-C motif ligand
CCR: C-C motif receptor
CNS: Central nervous system
CSF: Colony-stimulating factor
CXCL: C-X-C motif ligand
ctDNA: Circulating tumor DNA
DIPG: Diffuse intrinsic pontine glioma
DMG: Diffuse midline glioma
EZH2: Enhancer of zeste homolog 2
GBM: Glioblastoma
H3.1/3.3: Histone 3.1/3.3
H3K27M subtype: Histone 3 lysine 27 to methionine subtype
H3K27me3: Trimethylation of K27 of histone H3
IL: Interleukin
PDGFA: Platelet-derived growth factor subunit A
PDGFβ: Platelet-derived growth factor-beta
PD-L1: Programmed death ligand 1
pHGG: Pediatric high-grade glioma
pLGG: Pediatric low-grade glioma
PRC2: Polycomb repressive complex 2
SIRPα: Signal regulatory protein alpha
STI-1: Co-chaperone stress-inducible protein 1
TAM: Tumor-associated microglia/macrophage
TGF-ß: Transforming growth factor-beta
VEGF: Vascular endothelial growth factor
WHO: World Health Organization
